# 两个遗传性蛋白C缺陷症家系的临床特征与基因突变分析

**DOI:** 10.3760/cma.j.issn.0253-2727.2023.11.013

**Published:** 2023-11

**Authors:** 蔓霖 曾, 丽红 杨, 安庆 邹, 元 陈, 恺琦 贾, 明山 王, 艳慧 金

**Affiliations:** 温州医科大学附属第一医院医学检验中心，浙江省检验诊断及转化研究重点实验室，温州 325015 Department of Clinical Laboratory, the First Affiliated Hospital of Wenzhou Medical University, Key Laboratory of Clinical Laboratory Diagnosis and Translational Research of Zhejiang Province, Wenzhou 325015, China

蛋白C是一种由肝细胞合成和分泌的维生素K依赖性丝氨酸蛋白酶原[1]，是机体重要的生理性抗凝物质之一。蛋白C活化后能通过水解活化凝血因子Ⅴ（FⅤa）和活化凝血因子Ⅷ（FⅧa）来抑制凝血酶的产生。遗传性蛋白C缺陷症是由编码蛋白C的基因（PROC）突变引起的导致常染色体显性遗传疾病，纯合或复合杂合突变导致的严重蛋白C缺陷症极其罕见，发病率约为1/400万[2]，临床可表现为出生后不久发生的新生儿暴发性紫癜；单杂合型遗传性蛋白C缺陷症的发病率在普通人群中为0.14％～0.50％[3]，此类患者静脉血栓形成的风险可增加5～7倍。截至2021年4月，人类基因组突变数据库（HGMD）共列出391种PROC基因突变，其中错义/无义突变290种，小部分缺失突变30种。我们近期收治2例遗传性蛋白C缺陷症患者，并对其进行家系调查和基因突变分析。

## 对象与方法

1. 研究对象：调查2022年4月及2022年10月在温州医科大学附属第一医院就诊的两名遗传性蛋白C缺陷症先证者及其家系成员。询问有无血栓或出血病史，所有研究对象均进行蛋白C及相关抗凝蛋白水平和PROC基因检测。纳入同期100名健康体检者的外周血标本作为正常对照组，其中男56名、女44名，中位年龄32（16～60）岁，均无肝肾功能疾病，无血栓或出血史。所有受试者均签署知情同意书，本研究获得温州医科大学附属第一医院伦理委员会批准（KY2022-R193）。

2. 标本采集和DNA提取：经患者及家属知情同意后，采集患者及其家系成员外周静脉血2.7 ml，以0.109 mol/L枸橼酸钠1∶9抗凝，立即4 °C下1 000× *g*离心15 min，分离乏血小板血浆（PPP），分装后−40 °C保存，用于凝血指标检测；下层血细胞的基因组DNA由全血基因组DNA提取试剂盒（北京天根生化科技有限公司）提取，−40 °C保存待检。

3. 凝血指标检测：应用Stago-STA-R-Max全自动凝血仪（法国Diagnostica Stago公司产品）检测抗凝血酶活性（AT∶A）（发色底物法）、蛋白C活性（PC∶A）（发色底物法）和检测蛋白S活性（PS∶A）（凝固法）。采用酶联免疫吸附法检测蛋白C抗原（PC∶Ag）（试剂盒为温州长风生物技术有限公司产品）。

4. 凝血酶生成及抑制试验：使用校准的自动凝血酶生成方法（CAT）[4]检测凝血酶生成。正常血浆与受试者血浆在有无可溶性凝血酶调节蛋白（sTM）的情况下，评估凝血酶生成情况。根据制造商说明，使用FluoCa试剂盒（法国Stago公司产品）和20 µl PPP试剂，用5 pmol/L组织因子启动反应，进行CAT分析，最终形成凝血酶生成曲线。峰高（nmol/L）和内源性凝血酶电位（nmol/L*min）由凝血酶生成曲线推导。

5. PCR扩增和基因测序：引物由上海赛恒生物科技有限公司合成。在ABI Thermal cycler 2720（美国）扩增仪上扩增PROC基因所有9个外显子及侧翼系列。PCR产物送上海赛恒生物科技有限公司测序。使用Chromas软件与参考序列（GenBank: NG_016323.1）进行比对，查找基因突变位点。明确基因突变位点后，再扩增其家系其他成员相应突变位点区域并进行测序分析。此外，对先证者及其家系成员进行凝血酶G20210A突变和因子Ⅴ Leiden突变筛选。

6. 蛋白质生物学特性分析：应用在线生物信息学软件Mutation Taster（http://www.mutationtaster.org）预测基因突变的致病性。通过软件ClustalX-2.1-win将受突变影响的氨基酸与另外7种同源物种的氨基酸序列（https://www.ncbi.nlm.nih.gov/homologene/37288）进行比对，以分析突变氨基酸在物种进化过程中的保守性。

## 结果

1. 临床资料：家系1先证者，男，40岁，长途货车司机，2022年4月因连续驾车4 h后出现左下肢肿胀1周来我院就诊，入院后第2天出现咯血伴右侧胸痛。实验室检查示PC∶A 38％，PC∶Ag 40％，AT∶A 91％、PS∶A 92％，肝肾功能未见明显异常。双下肢动、静脉超声提示左侧肢深静脉血栓形成；肺动脉造影示右肺动脉主干及两侧肺动脉分支多发血栓形成，立即给予低分子肝素0.7 ml 每12 h 1次皮下注射抗凝治疗，9 d后改为利伐沙班15 mg每日2次直到症状好转，并建议出院后继续服用利伐沙班15 mg每日1次，为期3个月，然后调整为长期服用利伐沙班10 mg每日1次。家系1三代共7人纳入本研究，均否认血栓性疾病史。家系图见图1。家系成员抗凝血指标检测结果见表1。

**图1:**
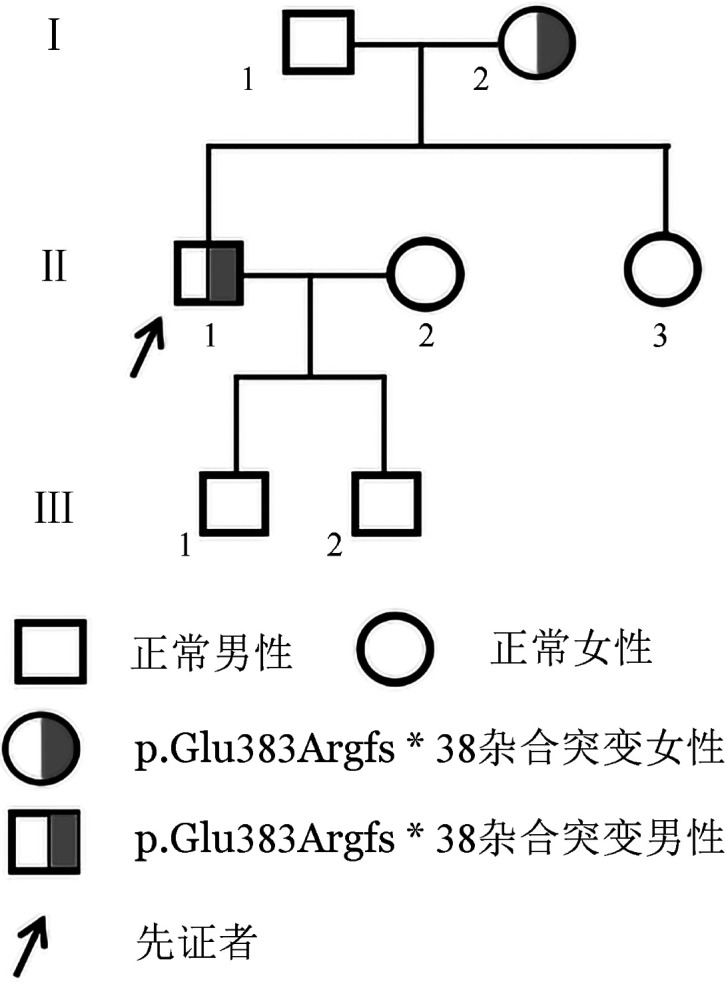
遗传性蛋白C缺陷症家系图（家系1）

**表1:** 遗传性蛋白C缺陷症家系（家系1）成员凝血指标检测结果（％）

家庭成员	PC∶A	PC∶Ag	PS∶A	AT∶A
先证者（Ⅱ_1_）	38	40	92	91
父亲（Ⅰ_1_）	102	110	93	100
母亲（Ⅰ_2_）	59	60	98	101
妻子（Ⅱ_2_）	105	109	88	100
妹妹（Ⅱ_3_）	108	121	80	105
长子（Ⅲ_1_）	107	120	95	108
次子（Ⅲ_2_）	104	115	90	104

参考值范围	70~130	70~140	65~135	98~119

注 PC∶A：蛋白C活性；PC∶Ag：蛋白C抗原；PS∶A：蛋白S活性；AT∶A：抗凝血酶活性

家系2先证者，男，30岁，公务员，无高血压、糖尿病和高脂血症病史，吸烟史10年，每天约20支。2022年10月因胸痛、晕厥于我院急诊就诊，实验室检查提示PC∶A 38％，PC∶Ag 78％，AT∶A 95％、PS∶A 87％，肝肾功能未见明显异常。冠状动脉造影示右冠状动脉开口及近端狭窄40％中段闭塞，行右冠状动脉抽吸术抽出大量血栓。术后予阿司匹林肠溶片100 mg每晨1次、氯吡格雷75 mg每晨1次、阿托伐他汀片20 mg每日1次、肝素钠6250 U微泵维持等治疗，后改以利伐沙班15 mg每日2次抗凝治疗，患者病情好转出院。3个月后，利伐沙班剂量调整为10 mg每日1次。家系2三代共5人，均否认血栓性疾病史。家系图见图2，家系成员凝血指标检测结果见表2。

**图2:**
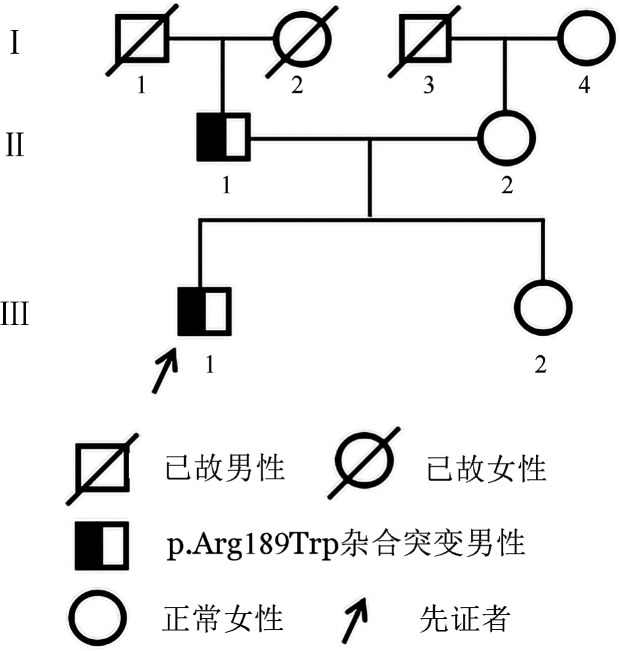
遗传性蛋白C缺陷症家系图（家系2）

**表2:** 遗传性蛋白C缺陷症家系（家系2）成员凝血指标检测结果（％）

家庭成员	PC∶A	PC∶Ag	PS∶A	AT∶A
先证者	38	78	87	95
外祖母	100	112	96	103
父亲	60	92	93	96
母亲	103	120	97	106
妹妹	99	107	90	100

参考值范围	70~130	70~140	65~135	98~119

注 PC∶A：蛋白C活性；PC∶Ag：蛋白C抗原；PS∶A：蛋白S活性；AT∶A：抗凝血酶活性

2. 凝血酶生成及抑制试验：与正常对照相比，两例先证者的内源性凝血酶电位和峰高均升高，其中家系1先证者升高较明显。在5 nmol/L sTM存在时，两例先证者的内源性凝血酶电位和峰高也较正常对照显著增加。与正常血浆中sTM介导的凝血酶生成抑制相比，两例先证者血浆对凝血酶生成的抑制能力均降低（图3）。

**图3:**
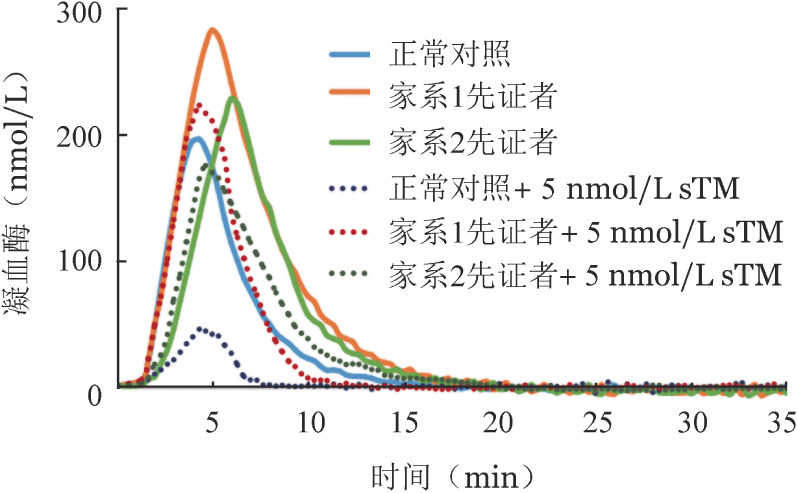
两个遗传性蛋白C缺陷症家系先证者的凝血酶生成及抑制试验曲线图（sTM：可溶性凝血酶调节蛋白）

3. 基因分析：家系1先证者PROC基因第9外显子存在c.1146_1146delT杂合缺失突变，导致p.Glu383Argfs*38，其母亲也存在c.1146_1146delT杂合缺失突变，其父亲、妹妹、妻子及两个儿子均为野生型，该缺失突变已行克隆测序验证。在千人基因组计划（https://www.genome.gov/27528684/1000-genomes-projec）中没有发现该突变，排除基因多态性的可能；查阅HGMD（https://www.hgmd.cf.ac.uk/ac/all.php）及相关文献中未见该突变的报道。家系2先证者PROC基因第7外显子存在c.565C>T杂合错义突变，导致p.Arg189Trp，其父亲也存在c.565C>T杂合错义突变，其外祖母、母亲及妹妹为野生型。在两名先证者或其家系成员中均未发现凝血酶原G20210A和因子ⅤLeiden突变。

4. 蛋白C生物信息学特性分析：Mutation Taster预测p.Glu383Argfs*38为致病突变，分数为1.000。保守性分析显示从Glu383到Val420这38个受突变影响的氨基酸在其7个同源物种中大多数位于保守区域（图4）。

**图4:**
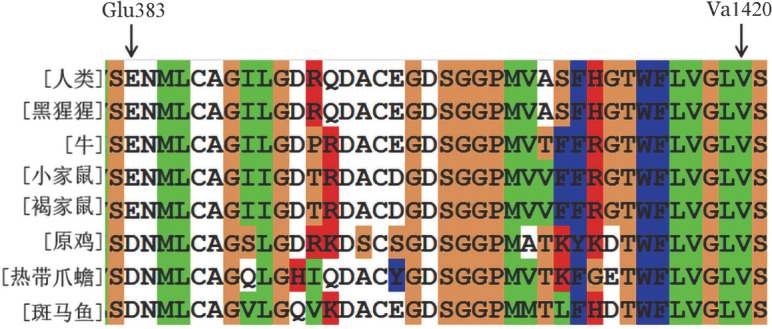
PROC基因同源物种保守性分析（箭头所示为突变氨基酸）

## 讨论

血栓栓塞症是由于遗传和环境因素等多种刺激因素之间的相互作用触发，遵从多重打击学说，机体主要的生理性抗凝物质（抗凝血酶、蛋白C和蛋白S）缺乏是亚洲人的重要危险因素[5]。蛋白C缺陷症的诊断主要依靠实验室对PC∶A水平和PC∶Ag含量的测定。在本研究中，两名先证者PC∶A水平均明显降低，基因突变分析显示分别存在p.Glu383Argfs*38杂合缺失突变和p.Arg189Trp杂合错义突变，属于遗传性蛋白C缺陷症。遗传性蛋白C缺陷症分为两类：Ⅰ型为PC∶A水平和PC∶Ag含量同步降低，Ⅱ型为PC∶A水平降低PC∶Ag含量正常。家系1先证者PC∶Ag含量同步降低，诊断为Ⅰ型遗传性蛋白C缺陷症；家系2先证者PC∶Ag含量正常诊断为Ⅱ型遗传性蛋白C缺陷症。凝血酶生成及抑制试验表明两例先证者血浆中的抗凝血活性均受损，其中家系1先证者表现为更明显。

蛋白C缺陷症患者经常出现复发性血栓事件，多以肺栓塞和（或）深静脉血栓形成为主。本研究家系1先证者为40岁男性，携带p.Glu383Argfs*38杂合缺失突变，合并肺栓塞和下肢深静脉血栓形成，实验室检查发现其PC∶A和PC∶Ag同步降低。有研究发现，蛋白C的催化结构域由第212～461位氨基酸组成，蛋白C的分泌需要依赖位于28个氨基酸环（398～426位）之后的羧基末端区域[6]。在本研究中，p.Glu383Argfs*38突变改变了自然阅读框，导致37个氨基酸（383～419位）发生替换和42个氨基酸（420～461位）完全丢失，这些氨基酸都位于PC的催化结构域，因此p.Glu383Argfs*38突变很可能影响了蛋白C的催化活性。另一方面，p.Glu383Argfs*38突变使得420位氨基酸编码提前终止，产生缺乏部分羧基末端氨基酸的截短蛋白，影响了蛋白C的分泌。此外，p.Glu383Argfs*38突变形成的截短蛋白非常不稳定，会在细胞内被加速降解，导致蛋白C水平降低。这与经Mutation Taster预测该突变为致病突变相符。因此，我们推测p.Glu383Argfs*38杂合缺失突变是家系1先证者发生遗传性蛋白C缺陷症的重要原因。该突变为新发现突变，致病性分类：①该突变为无功能突变能导致遗传性蛋白C缺陷症，属于非常强致病证据（PVS1）；②正常人群数据库中未发现该突变，属于中等致病性证据（PM2）；③该突变为缺失突变导致形成截短蛋白，属于中等致病性证据（PM4）。结合以上突变证据和分类，根据美国医学遗传学与基因组学学会（ACMG）遗传突变分类标准和指南，p.Glu383Argfs*38突变有1个非常强致病证据和2个中等致病性证据（1PVS1+2PM），评级为致病性突变。家系1先证者的母亲同为p.Glu383Argfs*38杂合突变携带，PC∶A为59％，既往却无血栓形成史，这可能与家系1先证者为长途货车司机存在长期久坐等易栓诱因有关。徐琦煜等[7]研究发现蛋白C缺陷症患者发生血栓的危险性随年龄增高而增加，当患者携带PROC突变并合并其他易栓危险因素（制动、吸烟、外伤、妊娠、口服避孕药等）时会进一步增加血栓栓塞的风险。这表明，p.Glu383Argfs*38突变与长期制动等获得性危险因素相结合时，会增加早期血栓形成的风险。

蛋白C缺陷症少数患者还可表现为动脉血栓形成，包括冠心病或心肌梗死和缺血性卒中等。Bereczky等[8]研究表明，蛋白C缺乏是年轻人动脉粥样硬化血栓形成疾病发病率的一个非常强的预测因子，蛋白C缺乏会使心肌梗死的风险增加8.76倍。本研究的家系2先证者为30岁男性，无高血压、糖尿病和高脂血症病史，有吸烟史，携带p.Arg189Trp杂合错义突变，其PC∶A降低、PC∶Ag正常，发生了冠心病。p.Arg189Trp杂合突变于1995年由Reitsma等[9]首次报道，也是中国人的热点突变[10]。p.Arg189Trp突变位点与PC轻链C端的表皮生长因子样结构域2（EGF-2）相邻，可能损害蛋白C与其他分子（如凝血酶血栓调节蛋白复合物、底物、PS或磷脂）的相互作用。杨丽红等[11]通过构建蛋白模型分析发现，p.Arg189Trp突变主要改变氨基酸侧链及氨基酸间的氢键连接和分子间作用力，从而影响蛋白质的正常折叠，并降低蛋白质结构的稳定性，形成结构异常的蛋白质，导致其生物学功能受影响。家系2先证者在年轻时发生动脉粥样硬化血栓形成疾病可能与其携带p.Arg189Trp杂合错义突变及吸烟等危险因素有关。在以往的报道中，携带p.Arg189Trp突变的患者均表现为静脉血栓形成[7],[9]–[11]，而同样的突变导致家系2先证者动脉血栓形成，目前国内外尚未见报道。

综上所述，本研究对2个遗传性蛋白C缺陷症家系的基因突变和临床特征进行了分析，首次报道了PROC（NM_000312）：c.1146_1146delT（p.Glu383Argfs*38）杂合缺失突变，并发现中国人群的突变热点c.565C>T（p.Arg189Trp）与动脉血栓形成也有关，其确切具体的致病机制需进一步研究。
